# New aQTL SNPs for the CYP2D6 Identified by a Novel Mediation Analysis of Genome-Wide SNP Arrays, Gene Expression Arrays, and CYP2D6 Activity

**DOI:** 10.1155/2013/493019

**Published:** 2013-10-22

**Authors:** Guanglong Jiang, Arindom Chakraborty, Zhiping Wang, Malaz Boustani, Yunlong Liu, Todd Skaar, Lang Li

**Affiliations:** ^1^Department of Medical and Molecular Genetics, Indiana University School of Medicine, Indianapolis, IN 46202, USA; ^2^Center for Computational Biology and Bioinformatics, Indiana University School of Medicine, Indianapolis, IN 46202, USA; ^3^Regenstrief Institute, Indianapolis, IN 46202, USA; ^4^Division of Clinical Pharmacology, Department of Medicine, Indiana University School of Medicine, Indianapolis, IN 46202, USA

## Abstract

*Background*. The genome-wide association studies (GWAS) have been successful during the last few years. A key challenge is that the interpretation of the results is not straightforward, especially for transacting SNPs. Integration of transcriptome data into GWAS may provide clues elucidating the mechanisms by which a genetic variant leads to a disease. *Methods*. Here, we developed a novel mediation analysis approach to identify new expression quantitative trait loci (eQTL) driving CYP2D6 activity by combining genotype, gene expression, and enzyme activity data. *Results*. 389,573 and 1,214,416 SNP-transcript-CYP2D6 activity trios are found strongly associated (*P* < 10^−5^, FDR = 16.6% and 11.7%) for two different genotype platforms, namely, Affymetrix and Illumina, respectively. The majority of eQTLs are trans-SNPs. A single polymorphism leads to widespread downstream changes in the expression of distant genes by affecting major regulators or transcription factors (TFs), which would be visible as an eQTL hotspot and can lead to large and consistent biological effects. Overlapped eQTL hotspots with the mediators lead to the discovery of 64 TFs. *Conclusions*. Our mediation analysis is a powerful approach in identifying the trans-QTL-phenotype associations. It improves our understanding of the functional genetic variations for the liver metabolism mechanisms.

## 1. Introduction

Genome-wide association studies (GWAS) have identified hundreds of genetic variants associated with complex human diseases, clinical conditions, and traits. These studies have also provided valuable insights into the genetic architecture. Unfortunately, GWAS studies have achieved limited success. The variants discovered usually explain only a small fraction of the overall heritability of the disease [1]. The identification of specific causal genes or mutations from associated regions is a challenge especially for the transacting SNPs which fall either far from genes or a region with many equally plausible causative genes. To make the situation more complicated, sometimes, a single locus can contain multiple independent risk variants (common or rare). Even when a locus is identified by SNP association, the causal mutation itself needs not to be a SNP [2]. For example, GWAS have associated the IRGM gene with Crohn's disease, but a subsequent study showed that the causal mutation is a deletion of the upstream of the promoter affecting tissue-specific expression [3].

There is a substantial gap in understanding the SNP traits associations from a genome-wide association study and the contribution of the locus to a disease. An eQTL approach investigates how the abundance of a gene transcript is directly modified by polymorphism in regulatory elements. The validity of eQTL has been shown in multiple tissue types, in which high heritability has been observed in widespread gene transcripts [4–8]. This indicates that genetic influences on gene expression are common. The potential of genome-wide eQTL identification has been shown originally in the yeast *Saccharomyces cerevisiae* [9] and then in humans, animals, and plants [10, 11]. One of the most important consequences of eQTL mapping is the link that it provides between genetic markers of a disease identified in GWAS and the expression of a specific gene or genes. In particular, the power of these studies depends upon the identification of specific genetic markers that are simultaneously associated with a disease and eQTLs. For example, a study generated genome-wide transcriptional profiles of lymphocyte samples from participants in the San Antonio Family Heart Study and showed that high density lipoprotein cholesterol concentration was influenced by the cis-regulated VNN1 [5, 12]. Another study of postmortem brain tissue identified eQTLs affecting the MAPT and APOE genes, which play an important part in Alzheimer's disease. Utilizing human lymphoblastoid cell lines from the HapMap project, recent pharmacogenomics study reveals novel genetic variants that contribute to etoposide-induced toxicity through affecting gene expression, which included genes that may play a role in cancer (AGPAT2, IL1B, and WNT5B) [13].

The substantial gap between associated regions from GWAS and the identification of causal variations that contribute to a disease might be filled by eQTL analysis. The functional effects of DNA polymorphism on a multifactorial disease can be mediated through several mechanisms. Polymorphisms responsible for the alteration in protein function can have important effects. However, systematic studies of complex diseases with known nonsynonymous SNPs have not yielded many highly significant results, and many associations implicate nonprotein coding regions. It has been shown that 5% of the human genome is evolutionary conserved and thus functional, whereas less than one-third of this 5% consists of genes that encode proteins [2]. Variation in gene expression is probably a more important mechanism underlying susceptibility to complex disease [2, 14].

Three major different methodologies have been developed and applied to the integrated eQTL and GWAS analyses. The first method focused on the overlapped SNPtrait, SNP-gene expression, and gene expression-trait associations [13, 15]. The second method employed the causal inference framework to identify causal model, reactive model, and independent model among SNP, gene expression, and traits. This approach brought in a more molecular mechanism in analyzing the data [16]. The third approach constructed a Bayesian network for the gene expression and traits, while the network construction was weighted by SNP-gene expression correlation [17].

A multistep procedure for identifying key driver of a complex trait has been described by Schadt et al. [16]. Pairwise regressions among genotype variation, gene expression, and complex trait are investigated first. Then the likelihood based causal model selection (LCMS) test is used to identify expression profiles that sit between the complex-trait QTL and complex trait. In this approach, without applying the statistical test for causality, three different models (causal model, reactive model, and independent model) are used. The particular model with the lowest AIC (Akaike information criterion) value is considered to be the best fit for the data. One great advantage of this procedure is that when a correlation between an expression trait and a clinical phenotype does exist, it can distinguish causal, reactive, or independent relationship between them.

### 1.1. Mediation Analysis

**Figure 1:**
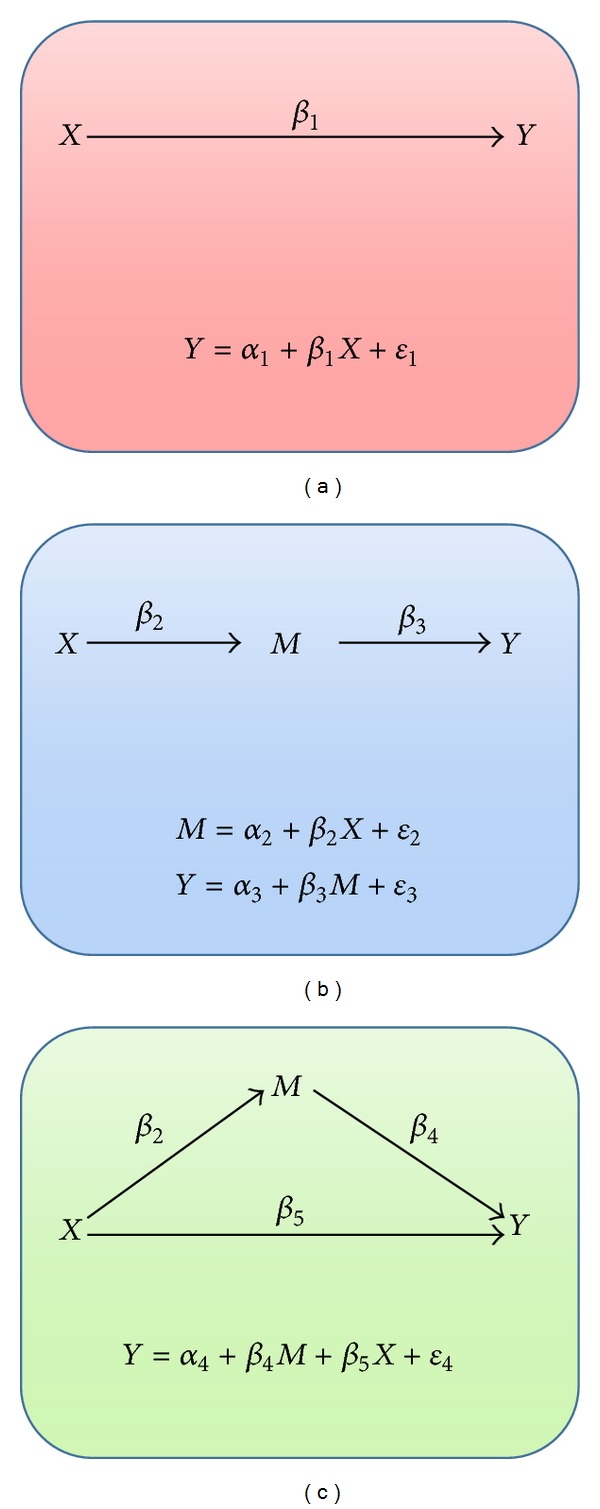
Mediation test.

Mediation analysis is the study of the causal chain or the indirect effect, to identify the possible underlying causal mechanisms. Mediation analysis is widely used across many disciplines such as social sciences, to identify the underlying causal mechanisms or to guide the experiments design [18]. A lot of research works focus on the relations between two variables, *X* and *Y*. Much has been written about two-variable relations, including conditions under which *X* can be considered a possible cause of *Y*. To this *X* → *Y* relation, one can add a third variable by using mediation, whereby *X* causes the mediator, *M*, and *M* causes *Y*, so *X* → *M* → *Y* (see Figure 1). If *X* leads to *Y* through *M*, this is called the *indirect effect*. Ignoring *M* leads to incorrect inference about the relation of *X* and *Y*, since the effect of *M* is confounded. If *M* is related to *X* and/or *Y*, so that information about *M* improves the prediction of *Y* by *X* but does not substantially alter the relation of *X* to *Y* when *M* is included in the analysis, then we consider *M* as a covariate. In another situation, *M* may also modify the relation of *X* to *Y* such that the relation of *X* to *Y* differs at different values of *M*. This is referred to as a moderator or interaction effect (see MacKinnon et al. [18] and references therein).

To establish this indirect relationship, Baron and Kenny [19] proposed a four-step approach in which several regression analyses are conducted, and the significance of the coefficients is examined at each step. In step 1, a simple regression analysis with *X* predicting *Y* is conducted (see Figure 1(a)) to test for path *β*_1_ as

(1) $$\begin{array}{c} Y=\alpha_{1}+\beta_{1}X+\varepsilon_{1}. \end{array}$$

In step 2, another simple regression analysis is performed with *X* predicting *M* to test for path *β*_2_ as

(2) $$\begin{array}{c} M=\alpha_{2}+\beta_{2}X+\varepsilon_{2}. \end{array}$$

And in step 3, the following regression equation is fitted with *M* predicting *Y* to test for path *β*_3_:

(3) $$\begin{array}{c} Y=\alpha_{3}+\beta_{3}M+\varepsilon_{3}. \end{array}$$

Step 2 and step 3 are combined in Figure 1(b). The final step is to conduct a multiple regression analysis with *X* and *M* predicting *Y* as (see Figure 1(c))

(4) $$\begin{array}{c} Y=\alpha_{4}+\beta_{4}M+\beta_{5}X+\varepsilon_{4}. \end{array}$$

In all the above steps, it is assumed that independently, *ε*_*k*_ ~ *N*(0, *σ*_*k*_^2^), *k* = 1,2, 3,4. The purpose of step 1–step 3 is to establish that zero-order relationships among the variables exist. One proceeds to step 4 assuming that there are significant relationships from steps 1 through 3. To identify potential causal links between genotype and clinical phenotypes, Huang et al. [20] designed a three-way model based on a stepwise regression analysis with genotype, gene expression, and cytotoxicity data as follows:

S1: SNP is associated with cytotoxicity,
S2: SNP is associated with gene expression,
S3: gene expression is associated with cytotoxicity.

Theoretical evidence in the form of “Causality Equivalence Theorem” has been proposed by Chen et al. [21] to establish causal relationship. According to the theorem, under the assumption that *X* is randomized, the following conditions are needed to establish a causal relation:

C1: *X* and *M* are associated,
C2: *X* and *Y* are associated,
C3: *X* is independent of *Y* | *M*.

If both *X* and *M* are significant predictors of *Y*, then *partial mediation* is achieved, whereas if *X* is no longer significant when *M* is controlled, this supports the condition of *full mediation*. However, there are some limitations of this test as mentioned by MacKinnon et al. [22]. This includes a low power to detect mediation and biased estimates. It does not test for the significance for the indirect pathway. An alternative and preferable approach to estimate the indirect effect is by multiplying two regression coefficients, *β*_2_ × *β*_4_ [23].

In this paper, we introduce a new method, mediation analysis, which is somewhere between the overlap analysis (the first method) and causal inference (the second method). We use the human liver consortium data to demonstrate its application and performance. We use genome-wide genotype and gene expression data to explore functional mutation for an important pharmacogene, CYP2D6, which is a member of the cytochrome P450 mixed-function oxidase system and is responsible for the metabolism of 25% of all drugs on the market.

## 2. Material and Methods

### 2.1. Human Liver Cohort Dataset

Human liver cohort (HLC) data are collected from Sage Bionetworks Repository and Gene Expression Omnibus (GEO) database as described in the literature [17]. The dataset includes 2 genotype arrays (Illumina Sentrix human Hap650Y genotyping beadchip and Affymetrix 500 K genotyping array), gene expressions (30,128 probes × 466 samples) and enzyme activities (10 activity measurements of 9 enzymes × 488 samples), and demographic information. Genotype data for 219 Illumina and 214 Affymetrix that are publicly accessible are used. Patients with genotyping call rate less than 95% are removed from further analysis. This filtration reduces the sample sizes to 204 and 207 for Affymetrix and Illumina platforms, respectively. 167 Illumina genotyping has both gene expression data and enzyme activity data. In case of Affymetrix platform, 180 samples overlapped with gene expression and enzyme activity data.

SNPs whose genotyping call rate are less than 95% or Hardy-Weinberg equilibrium tests are significant (*P* < 0.001) or minor allele frequency <10% are discarded. For Affymetrix platform, 214,399 SNPs, and for Illumina, 471,394 SNPs are used for mediation and eQTL analysis. Enzyme activity and gene expression data are corrected with age and gender and then are normalized with normal quartile normalization.

### 2.2. Mediation Analysis

The mediation analysis method is developed to assess the indirect effects of genetic variant to CYP2D6 activity mediated by gene expressions. The tests are performed by parallel programming using C and MPICH. The computations are run on a Linux cluster computing environment with 200 compute nodes, and each node takes around 36 hours.

MacKinnon proposed a permutation test for mediation that makes use of the permutation-of-raw-data approach for testing a regression coefficient [22, 24]. It is referred to as the *permutation test of* *β*_2_ × *β*_4_. To test for regression coefficients, permutation tests have been applied in several ways [24–26]. Applying this method requires, first, that the regression models in (2) and (4) are estimated for the original, nonpermuted data to find the values of *β*_2_  and  *β*_4_. Values of the outcome variable, *Y*, are then permuted 10^9^ times and reassigned to nonpermuted scores on the predictor, *X*, and mediator, *M*, to create many permuted samples. The permuted *Y* values, labeled *Y* ^+^, are then regressed on the nonpermuted *X* and *M* values in each permuted sample (as in (4)), and the coefficient for *M* in each permuted sample is labelled *β*_4_*. Similarly, values of the mediator, *M*, are permuted 10^9^  times and reassigned to values of the predictor *X* to create many permuted samples. The permuted *M* values, labeled *M* ^+^, are regressed on *X* in each permuted sample (as in (2)), and the coefficient for *X* in each permuted sample is labelled  *β*_2_^+^. Finally, corresponding pairs of *β*_2_^+^ and *β*_4_^+^ values are multiplied to yield *β*_2_^+^ *β*_4_^+^, and ${\hat{\beta }}_{2}{\hat{\beta }}_{4}$, the estimate of the mediated effect from the original data, is compared to the distribution of *β*_2_^+^ *β*_4_^+^ to perform a test of the null hypothesis of no mediation.

The mediated effect is estimated by the product of coefficients (*β*_2_ × *β*_4_) then divided by its standard error, which is derived by Sobel [23], under the assumption of multivariate normality for the standard error of the indirect effect, using the multivariate delta method as

(5) $$\begin{array}{c} \sigma_{\beta_{2}\beta_{4}}=\sqrt{\beta_{2}^{2}\sigma_{4}^{2}+\beta_{4}^{2}\sigma_{2}^{2}}. \end{array}$$

Hence, the test statistics are

(6) $$\begin{array}{c} \Delta_{\text{Indirrect}\;\text{effect}}=\frac{{\hat{\beta }}_{2}{\hat{\beta }}_{4}}{\text{se}\left({\hat{\beta }}_{2}{\hat{\beta }}_{4}\right)}. \end{array}$$

### 2.3. Genome-Wide Association Based on Mediation Analysis

The huge sizes of SNP and gene expression probes in mediation analysis introduce problems related to multiple hypotheses testing. False discovery rate (FDR) is used to control type I error for multiple testing. FDR is calculated as

(7) $$\begin{array}{c} \text{FDR}=\frac{\#\;\text{significance}\;\text{by}\;\text{chance}}{\#\;\text{significance}\;\text{results}}. \end{array}$$

A stringent threshold is needed to avoid high FDR. Comparing to cis-acting variations, more transacting variations are detected by GWAS. In GWAS analysis, transeffects are usually weaker than cis-effects but are more numerous than the latter [14]. The trans-acting SNPs having smaller effects than cis-acting SNPs are more likely to be missed if more stringent threshold is applied.

### 2.4. eQTL Analysis

Transcript abundance is highly heritable in human populations and can be considered as a quantitative trait and be mapped to particular genomic loci, known as expression quantitative loci (eQTL). Not only gene expression is itself a complex trait, but also it acts as an intermediate phenotype between genetic loci and higher level cellular or clinical phenotypes, such as disease risk or individual drug response [27].

Linear model is fitted with genome-wide genotype and gene expression profiles. eQTL analysis is run in parallel on the same computing cluster with R language program. eQTL hotspots are defined as SNPs enriched in correlations with expression profiles across the genome (SNPs correlated with at least 20 gene expression profiles). The correlation *P* values between SNP and expression probe less than 10^−5^ are considered to be significant and used for hotspot analysis. To test the enrichment of significant correlation between eQTL and all gene expression probes, exact binomial tests are conducted and corrected with Bonferroni method, and the corrected *P* values are used as the enrichment scores.

## 3. Results

### 3.1. Mediation Analysis

The result of mediation analysis is summarized in Table 1. To find the significant trios, *P* values less than 10^−5^ are considered. Using the same criteria for both platforms, the number of significant trios differs. For Affymetrix platform, we have 389,573 trios having *P* values less than 10^−5^. For the other platform, this number is 1,214,416. The FDR for Illumina platform is found to be 11.73%, whereas for Affymetrix platform it is a bit higher (16.63%).

### 3.2. eQTL Analysis

**Figure 2:**
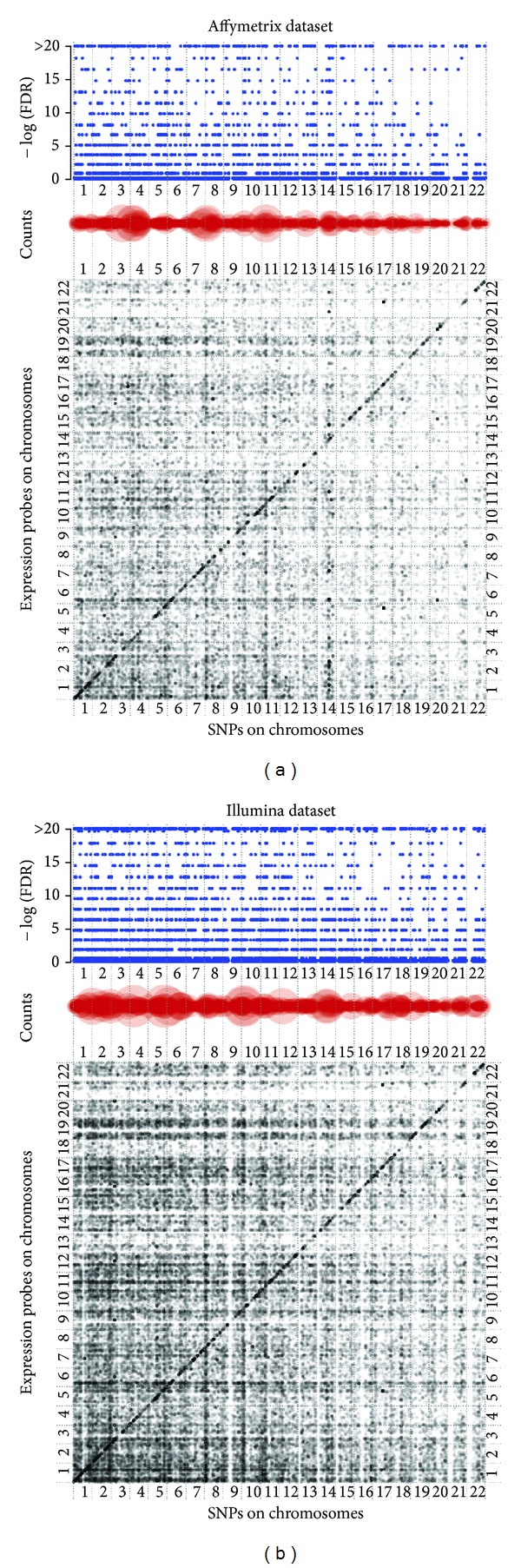
eQTL visualization. The main plot at the bottom is the scatter plot of the eQTL-transcript association. Each dot denotes a significant association between a SNP and a transcript (*P* value <10^−5^). Gray color shows the level of significance where dark means more significant association. SNPs are arranged according to their chromosomal loci along the *X*-axis from chromosome 1 to 22, and genes are arranged along *Y*-axis in the same way. The dots along diagonal line indicate cis-eQTLs, otherwise, trans-eQTLs. The counts plot in the middle gives the number of genes that a SNP correlated with significantly (*P* value <10^−5^). Large size means more genes associated with that SNP. The −log_10_ (FDR) plot at the top presents the enrichment score of a SNP associated with multiple transcripts comparing with that by chance. SNP has a large circle in counts plot and a high enrichment score in −log_10_ (FDR) plot which indicates eQTL hotspots.

In Table 2, the result corresponding to eQTL analysis of the HLC data is reported. The Affymetrix dataset has 214,399 SNPs after the implementation of the quality control out of which 28,089 are correlated with at least one gene at *P* < 10^−5^ significance level, and there are total 65,763 SNP-gene pairs significantly correlated. 295 SNPs are correlated with at least 20 genes. Those 295 hotspots are used to check for overlapping with the results of mediation analysis. 289 eQTL hotspots are found correlated with 1542 gene expression profiles at *P* < 10^−5^ significance level (Table 3). In contrast, Illumina dataset has higher quality with more SNPs passed quality control tests. Out of 471,394 SNPs, 63,643 SNPs are found to be correlated with at least one gene at *P* < 10^−5^ significance level. Numbers of SNPs that are correlated with at least 20 genes are found to be 724, and 719 of the hotspots are significantly correlated with 2,444 genes in mediation analysis (Table 3). In Figure 2, a pictorial depiction of this eQTL analysis is given for both platforms. The significant SNP-expression pairs (*P* < 10^−5^) are plotted as a dot according to the locations of the SNP and the gene on 22 chromosomes along *X*-axis and *Y*-axis. The grey colors show the level of significance, with darker dots representing smaller *P* values. The counts of significant SNP-expression pairs and −log_10_ (FDR) for a given SNP are also plotted above the eQTL image. For each SNP, the count gives the number of genes that are correlated with this particular SNP, as the larger radius of the circle indicates that the SNP is correlated with more genes. In that case, it may be considered to be a potential eQTL hotspot. The dots along diagonal line indicate cis-effects. It can be seen that cis-eQTLs have bigger effect on expression profile compared to trans-eQTLs.

### 3.3. Functional Analysis of Hotspots Mediators

1,542 and 2,444 hotspot mediators from Affymetrix and Illumina platforms annotated to 1,388 and 2,187 unique genes separately. 939 and 1420 genes are successfully mapped in Ingenuity database for two platforms. The functional annotations of these genes are summarized in Table 4. Five (CCL16, CCL20, CMTM5, IL6, and SPP1) and 7 (CCL16, CCL20, CKLF, CKLFSF5, EPO, FAM3C, and SPP1) cytokines, 5 (AR, NR1I2, NR1I3, NR2F6, and PPARA) and 7 (AR, ESR1, NR1I2, NR1I3, PPARA, RORA, and RORC) ligand-dependent nuclear receptors, and 80 and 113 transcription regulators are found to mediate the relationship between genetic variant and CYP2D6 activity for Affymetrix and Illumina platforms. 64 transcription regulators overlapped between the two platforms (Gene List 1). Among the 64 transcription factors predicted mediateding genetic regulation of CYP2D6 activity, YY1 is reported putatively binding to gene CYP2D6 promoter region and regulating the expression of CYP2D6 and CYP2D4 [28, 29].

## 4. Conclusion

Cytochrome P450 constitutes a large subfamily of enzymes that plan an important role in the metabolism of endogenous compounds and the activation of chemical carcinogens. In this work, the regulations of P450 expression and activities have been intensely studied. Several other studies have found that P450 are subject to regulation by liver-enriched transcription factors, cytokines, and nuclear receptors. Our study provides some new clues on the regulation of CYP2D6 enzyme activity. Our mediation analysis is a powerful approach in identifying the trans-SNP-phenotype associations. We found a rich class of functional categories of mediators that potentially control the CYP2D6 activities, which include many new transcription factors. This method has some limitations too. In this work, the relationship between genetic variants, gene expression, and phenotype is assumed to be a simple one. However, in most of the situations, this relationship may become very complex. More sophisticated methods are required to analyze those complex models. In mediation analysis, we are only interested in testing the product of two regression coefficients. Mediation analysis cannot provide causal inference. The mediation analysis assumes that there is some causal relationship. It will be necessary to test for the assumption. We need to be extra cautious about drawing the conclusion of the causal relationship. Our studies provide insights into the comprehension of the complex regulatory network of CYP2D6 and improve our understanding of the functional genetic variations for the liver metabolism mechanisms.

## 5. Genes List

64 TFs overlapped between Affymetrix and Illumina datasets, including AATF, ALYREF, ARHGAP35, ASB8, ATF4, CBX4, CEBPG, CSDA, DDIT3, E2F5, ETV7, FOXN3, FOXN3, FUBP1, GPS2, HDAC10, HMGN1, ID1, INVS, IRF9, KANK1, KAT2B, KHDRBS1, KLF12, MAF, MAML2, MEIS2, MLXIPL, MXD4, MYBBP1A, MYCL1, NCOA7, NCOR1, NFIA, NFKB2, NFYA, NOLC1, NPM1, PEX14, PYCARD, SAP18, SATB1, SIM2, SLC2A4RG, SMARCC1, SNAI3, SNW1, SOX5, TCERG1, TCF7L2, TEAD3, TEAD4, TFDP2, TFEB, TOB1, TP53, YWHAB, YY1, ZGPAT, ZHX3, ZKSCAN1, ZNF132, ZNF256, and ZNF263.

## Figures and Tables

**Table 1:** Mediation analysis.

Genotype dataset	Enzyme	SNP effect	No. of sig. trios *P* < 10^−5^	No. of sig. SNPs *P* < 10^−5^	No. of sig. exp *P* < 10^−5^	FDR (trios)
Affymetrix	CYP2D6	Gene dose	389,573	103,369	3,545	16.63%
Illumina array	CYP2D6	Gene dose	1,214,416	251,738	4,770	11.73%

**Table 2:** eQTL analysis.

Genotype dataset	No. of pairs *P* < 10^−5^	No. of SNPs Correlated with >1 gene (total SNPs)	No. of SNPs Correlated with >20 genes
Affymetrix	65,763	28,089 (214,399)	295
Illumina	154,546	63,643 (471,394)	724

**Table 3:** QTL overlapping.

Overlapping	Affymetrix		Illumina	
	No. of eQTL hotspots	No. of mediation trios	No. of eQTL hotspots	No. of mediation trios
	295	389,573	724	1,214,416
No. of eQTL hotspot trios (No. of SNPs, No. of genes)	9,296 (289, 1,542)		34,880 (719, 2,444)	

**Table 4:** Functional annotations of the mediators.

Types	Affymetrix no. Mediator genes	Illumina no. Mediator genes
Cytokine	5	7
Enzyme	246	368
G-protein coupled receptor	17	20
Growth factor	5	11
Ion channel	13	18
Kinase	52	62
Ligand-dependent nuclear receptor	5	7
Other	373	596
Peptidase	31	39
Phosphatase	15	21
Transcription regulator	82	118
Translation regulator	6	10
Transmembrane receptor	12	16
Transporter	77	127

Sum	939	1420

## Abbreviations

GWAS: Genome-wide association study
eQTL: Expression quantitative trait loci
aQTL: Enzyme activity quantitative trait loci
FDR: False discovery rate
HLC: Human liver cohort
TFs: Transcription factors.
